# Live Birth Rates After Varicocele Embolization in Patients After Failed In Vitro Fertilization With Intracytoplasmic Sperm Injection

**DOI:** 10.1111/andr.70328

**Published:** 2026-08-13

**Authors:** Anthony Verstandig, Adam Farkas

**Affiliations:** ^1^ Interventional Radiology Unit Shaare Zedek Medical Center Jerusalem Israel

**Keywords:** assisted reproductive techniques, in vitro fertilization, intracytoplasmic sperm injection, male factor infertility, varicocele embolization, varicocele repair

## Abstract

**Background and Objectives:**

While in vitro fertilization with intracytoplasmic sperm injection (IVF‐ICSI) bypasses abnormalities in sperm concentration, motility, and morphology, it still has a significant failure rate. We report our experience on the effect of varicocele embolization (VE) on couples with varicocele related primary male factor infertility in whom IVF/ICSI failed to result in a live birth (LB).

**Material and Methods:**

This single center retrospective cohort study documents 42 couples who had failed to have a LB after single or multiple IVF/ICSI cycles and in whom the male partner subsequently had left or bilateral varicoceles treated by transcatheter embolization. Other inclusion criteria were maternal age of 35 or less at the time of VE and at least 24 months follow up after embolization. The end point was LB by natural conception or by IVF‐ICSI.

Pre‐procedure data was documented at the time of referral for varicocele embolization. Images of the procedures were reviewed, and post VE data was collected by telephone questionnaire from one of the partners.

**Results:**

Prior to varicocele embolization, the 42 couples had undergone 151 oocyte retrievals (median 2, range 1–21) and 200 embryo transfers (median 3, range 1–26) with no LB.

During a median follow up period of 54 months (range 25–111) following VE, there were 60 LB (65 children) amongst 36 of the 42 couples (86%). Thirty‐four couples underwent a total of 119 oocyte retrievals and 149 embryo transfer procedures with 28 achieving 36 LB (41 children). Fifteen couples had 23 LB (23 children) from natural conceptions. One couple had an LB after intrauterine insemination.

**Conclusion:**

VE in this challenging patient population may treat varicocele associated sperm function problems not addressed by ICSI and could be beneficial before assisted reproductive techniques in varicocele related male factor infertility.

## 1 Introduction

Infertility is defined as the inability to conceive after 1 year of unprotected intercourse and is reported to occur in up to 15% of couples [1]. Male factor infertility (MFI) is solely responsible for 20%–30% of infertility cases but contributes to up to 50% of cases overall [1, 2].

Varicocele is the most common treatable causes of MFI with an incidence of 15% in all males rising to 35%–40% in men with primary infertility and up to 80% in men with secondary infertility [3, 4].

Varicoceles are dilated scrotal veins occurring when there is dysfunction or absence of the one way valves normally present in the long vertical spermatic veins. This allows the flow of warm blood, under increased hydrostatic pressure, from the abdomen to the testicles. Varicocele is associated with heat stress and excess reactive oxygen species which can affect standard semen parameters and the integrity of the sperm DNA [5]. It can also disrupt many aspects of sperm function including sperm–egg recognition, acrosomal exocytosis, and sperm–oocyte fusion [6].

Several meta‐analyses have demonstrated beneficial effects of varicocele repair (VR) on sperm parameters [7, 8, 9], DNA integrity [10, 11, 12], spontaneous pregnancy (SP), live birth rates (LBR) [13, 14, 15], testicular sperm extraction rates [16, 17], and results after assisted reproductive techniques (ART) [18, 19]. VR has not, however, gained widespread acceptance, due in part to the lack of sufficiently powered well‐designed trials to conclusively demonstrate its effect on LBR. Most severe MFI is treated by in‐vitro fertilization usually with intracytoplasmic sperm injection (IVF‐ICSI) often without full investigation of the male [20], as recommended in the 2024 updated guidelines of the American Urological Association and American Society for Reproductive Medicine [21]. This can result in failure to identify potentially reversible causes such as varicocele. While IVF/ICSI is effective, the Center for Disease Control report for ART in 2022 reported that only 37.5% of ART cycles resulted in a LB with many couples therefore requiring more than one cycle [22].

In 1989 Ashkenazi et al. reported their results in 10 couples with isolated MFI who had previously failed IVF, achieving a 40% pregnancy rate (PR) after VR [23]. Similarly, Yamamoto et al. reported 13 couples with normal female fertility, severe oligozoospermia and varicocele who had failed IVF and achieved a 31% PR after VR [24]. No studies have reported the results of VR on couples who have failed IVF/ICSI.

The purpose of this study is to present our experience in 42 couples who underwent VR by transcatheter embolization after failed IVF/ICSI for MFI.

## 2 Materials and Methods

Approval for this retrospective study was granted by the hospital Helsinki Ethics Committee (Approval No. 0335‐21‐SZMC). No written consent was obtained from the patients as there is no patient identifiable data included in this case series. Pre‐procedural outpatient clinic records for all the patients who underwent varicocele embolization at a single center between May 2013 and December 2019 were reviewed. All couples who had undergone one or more IVF/ICSI cycles not resulting in a live birth (LB) or ongoing pregnancy before the clinic visit were identified. Couples with azoospermia, or maternal age of more than 35 were excluded. The remaining patients comprise the study group. The primary end point was LBR after transcatheter embolization of varicocele where LBR is defined as the percentage of the study cohort who had live births during the follow up period.

Pre‐procedure data documented at the initial clinic visit included the duration of infertility prior to VR, spermogram results, female infertility factors and the number of IVF/ICSI cycles, and embryo transfers (ET). ET refers to the number of embryo transfer procedures, some involving transfer of more than one embryo. The procedural radiology images were reviewed to determine whether embolization was unilateral or bilateral.

Post procedure data, collected by a phone interview with the male or female partner, included the number of IVF/ICSI cycles and ET post varicocele embolization (VE), natural or IVF/ICSI births, and time from the VE procedure to the first birth.

Patients were referred to the varicocele clinic based on duplex evidence of varicocele variously reported as “varicocele present”, “vein diameter in mm”, “presence of reflux” or as duplex varicocele grading. The decision to treat was based on the presence of abnormal semen parameters and a palpable varicocele in accordance with American Urological Association/American Society for Reproductive Medicine [21] and European Association of Urology guidelines [25].

Clinical examination was performed with the patient standing and performing the Valsalva maneuver or coughing. In cases with unclear findings, the examination was repeated after 30–60 min of standing. All palpable varicoceles, constituting Amelar and Dubin Grades 1–3, were treated [26]. Right sided varicoceles were frequently not detected on duplex ultrasound but were detected or suspected on clinical examination in some patients. In these patients, the confluence of the right spermatic vein with the inferior vena cava was catheterized. Right varicocele was diagnosed and treated if there was free passage of injected contrast to the inguinal canal during a Valsalva maneuver or if there were significant retroperitoneal veins draining into the spermatic vein caudal to a patent valve.

*Procedural technique*: All patients were treated from the right internal jugular vein approach with embolization coils (Cook Inc., Bloomington, IN, USA) placed at the level of the inguinal ligament and 3% sodium tertradecyl sulfate (STD pharmaceutical products Ltd., Hereford, England, UK) foamed with an equal volume of air as a sclerosant injected above. Additional coiling was then performed more cranially in the vein. This sandwich technique has been previously described and is designed to provide permanent occlusion of both the main spermatic vein and potential collaterals that could result in recurrence, using a minimal radiation dose [27].

## 3 Results

During the study period, 1013 technically successful VE were performed. This included 59 couples who had undergone unsuccessful IVF/ICSI treatments prior to the embolization. Seventeen were excluded for the following reasons: inability to contact (*n* = 2), age of female partner more than 35 at time of VE (*n* = 12) or failure to undergo IVF post VE despite lack of SP (*n* = 3). The remaining 42 couples who had undergone 151 oocyte retrievals (median 2, range 1–21) and 200 ET (median 3, range 1–26) with no LB constituted the study group. In Israel with universal coverage of fertility procedures for childless couples, all cases of significant male infertility treated by IVF will have ICSI as part of the procedure to maximize success rates. Demographic data and pre‐embolization IVF/ICSI treatments are reported in Table 1. VE procedures were technically successful in all patients with 21 on the left only and 21 bilateral and with no reported complications.

**TABLE 1:** Demographic and pre‐varicocele embolization data.

Median age at time of VE	male	31 (range 23–37)
	female	27 (range 22–34)
Median period of infertility before VE		48 months (range 23–156)
Significant female infertility factor *n* = 5		
Total motile sperm count^a^		
	< 2 million	n = 32
	>2 to <5 million	*n* = 5
	>5 to <20 million	*n* = 4
Pre‐varicocele embolization ART procedures		
Ovarian stimulation cycles^b^ (*n* = 151) median 2.5/couple (range 1–21)		
Embryo transfers (*n* = 200) median 3/couple (range 1–26)		
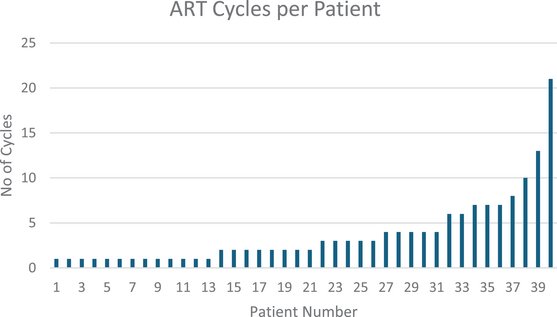		
Complications from IVF/ICSI procedures.		
Extrauterine pregnancy (*n* = 5)		
Hospitalization for ovarian hyperstimulation syndrome (*n* = 1)		

^a^ Data missing for 1 patient.

^b^ Data missing for 2 patients.

During a median follow‐up period of 54 months (range 25–111), there were 60 LB (65 children) amongst 36 of the 42 couples (86%). Median time from VE to the first pregnancy was 15.5 months (range 2–81). Thirty‐four couples underwent a total of 119 oocyte retrievals and 149 ET procedures. Twenty‐eight of these couples achieved 36 LB (41 children). Fifteen couples had 23 LB from SP including 6 subsequent to LB from IVF/ICSI. One couple had an LB from intrauterine insemination (IUI).

At the time of data collection, there were 7 ongoing pregnancies of more than 23 weeks, 5 from IVF/ICSI and 2 by SP. Details of the number of LB per couple and how they were achieved are given in Table 2.

**TABLE 2:** Live births after varicocele embolization and technique of conception.

Live births/couple	No. of couples	Total live births	Births by IVF	Births by natural conception	Births by IUI
0	6	0	0	0	0
1	22	22	16	5	1
2^a^	8	16	12	4	0
3^b^	3	9	4	5	0
4	2	8	4	4	0
5	1	5	0	5	0
TOTAL	42	60	36	23	1

^a^ Four patients with initial IVF births had subsequent births from natural conception.

^b^ Two patients with two births from IVF conceptions had a third from natural conception.

Subgroup analysis according to the number of pre‐VE IVF/ICSI cycles was performed (data missing for 2 couples). Twenty‐two of the 26 couples who underwent 3 or fewer cycles (median = 1.5) had live births (85%). Twelve of the 14 couples who underwent 4 or more cycles (median = 6.5) had live births (86%).

## 4 Discussion

In this study, 42 infertile couples who had failed to achieve LB after 151 oocyte retrieval cycles with 200 ET, underwent VE. During a median follow‐up period of 54 months there were 60 live births amongst 36 (86%) couples confirming that VR is effective in this challenging group of patients. This is the first study documenting the effect of VR on patients after failure of IVF/ICSI, with each couple acting as their own control. It should be noted that these results were in a cohort of couples with the female partner being aged 35 or less and should not be assumed to apply to older females.

The 36% LB rate resulting from SP is consistent with reports in the literature. The 2021 Cochrane report on the efficacy of VR in subfertile men reported that in 7 randomized controlled trials including 693 patients with abnormal sperm parameters and clinical varicocele, “VR may improve spontaneous pregnancy rates compared to delayed or no treatment (RR 1.94, 95% CI 1.23 to 3.05; *I* ^2^ = 61%,). This suggests that couples without treatment or delayed treatment have a 21% chance of pregnancy, whilst the PR after surgical or radiological VR is between 26% and 64%” [15].

In a 2019 meta‐analysis by Ou et al. of studies involving infertile patients with bilateral varicocele, bilateral repair was shown to result in more SP than unilateral treatment (OR 1.89. 95% CI 1.52–2.35; *I* ^2^  = 0%; *p* < 0.00001), justifying the treatment of bilateral varicoceles in appropriate patients [28]. In a 2025 meta‐analysis of 5 studies involving 746 couples with varicocele related MFI undergoing ART, (348 who underwent VR and 398 controls) Teng et al. reported the LBR was significantly higher in the VR group (OR: 2.18, [1.58, 3.01]; *p* < 0.00001: heterogeneity; *I* ^2^ = 0%) [19]. The mechanism for this is unclear but it appears that while ICSI bypasses shortcomings in sperm concentration, motility and morphology, there are varicocele related sperm function factors that are not repaired by this procedure. These factors could include sperm DNA fragmentation and failure of oocyte activation due to low expression of Phospholipase C‐zeta, both known to be adversely affected by the presence of varicocele and to cause decreased fertilization, implantation, and embryo development [29].

Incorporating VR in the treatment of varicocele associated MFI could decrease the required number of ART procedures resulting in increased patient safety and decreased costs. This decrease in the number of procedures could be a result of SP, increase of the LBR per IVF/ICSI cycle, or increase of the total motile sperm count resulting in couples becoming eligible for IUI [30, 31].

### 4.1 Safety

Reducing the number of IVF/ICSI procedures would reduce the complications to the mother and offspring such as those associated with ovarian hyperstimulation syndrome, ectopic pregnancy, multiple births, and the significant increase in the relative risk of birth defects [32, 33, 34]. In contrast, VE is a minimally invasive procedure with a low rate of significant complications [35].

### 4.2 Cost

ART is expensive, involving the costs of the procedures, associated medications, monitoring studies, and lost workdays as well as those of managing complications. These expenses may be incurred for every cycle. VR by comparison is a relatively inexpensive procedure required just once as, unlike ART, it treats the root cause of the infertility problem and should be effective for all desired future births so that a relatively small expense may result in a considerable overall cost reduction [36, 37].

There are several reasons why VR for MFI is not routinely practiced. Firstly, the literature on the efficacy of VR is not conclusive due to underpowered and heterogeneous studies. Secondly, the great success of IVF/ICSI in achieving LB with even a single sperm cell has resulted in a tendency to proceed directly to this treatment in severe MFI often without fully investigating the male partner with scrotal physical examination or duplex ultrasound to detect varicocele [38]. Thirdly, by the time couples reach the stage of ART, often without VR having been considered, they are often seeking a quick solution. IVF/ICSI holds out the hope for this quick solution so that the prospect of a VR procedure that may require 3 months to improve sperm parameters, is comparatively less attractive.

## 5 Weaknesses

This study has no control group so it is unknown how many LBs could have resulted from continued IVF/ICSI without VR. Large studies, however, have demonstrated a progressive decline in both the optimal and conservative estimates of the cumulative LBR with increasing cycle number [39]. Our study cohort had failed a median of 2 IVF/ICSI cycles prior to VE so the most optimistic estimate of LBR from subsequent cycles would be far below the 86% achieved in this study. This is even more pronounced in the 18 couples after 4 or more failed IVF/ICSI cycles who achieved an 86% LBR. The study is retrospective relying on data from patients’ memories, adding a possible degree of inaccuracy to some of the data. Some of the couples underwent IVF/ICSI treatments shortly after VR without waiting for possible SP, so that the number of natural births that could have resulted from VR may be even larger than that attained. This is suggested by the 6 couples in whom IVF/ICSI births were succeeded by natural births.

## 6 Conclusion

These results support the efficacy of VR, either alone or as an adjunct to IVF/ICSI, in this challenging cohort of patients and suggest that there are varicocele‐associated defects in sperm function not overcome by IVF/ICSI. Couples with MFI should be investigated to detect clinical varicoceles and counseled as to the potential contribution of VR to attaining LB either as a stand‐alone treatment or in combination with ART.

## Author Contributions

Anthony Verstandig conceived the topic, collected and analyzed the data and performed many of the procedures. Adam Farkas performed many of the procedures and significantly contributed to the data analysis and presentation.

## Funding

The authors have nothing to report.

## Conflicts of Interest

The authors declare no conflicts of interest.

## Data Availability

The data that support the findings of this study are available from the corresponding author upon reasonable request.
